# Type 2 diabetes is associated with loss of HDL endothelium protective functions

**DOI:** 10.1371/journal.pone.0192616

**Published:** 2018-03-15

**Authors:** Tomáš Vaisar, Erica Couzens, Arnold Hwang, Michael Russell, Carolyn E. Barlow, Laura F. DeFina, Andrew N. Hoofnagle, Francis Kim

**Affiliations:** 1 UW Medicine Diabetes Institute, Department of Medicine, University of Washington, Seattle, Washington, United States of America; 2 The Cooper Institute, Dallas, Texas, United States of America; 3 Department of Laboratory Medicine, University of Washington, Seattle, Washington, United States of America; South Australian Health and Medical Research Institute, AUSTRALIA

## Abstract

**Aims/Hypothesis:**

One of the hallmarks of diabetes is impaired endothelial function. Previous studies showed that HDL can exert protective effects on endothelium stimulating NO production and protecting from inflammation and suggested that HDL in obese people with diabetes and dyslipidemia may have lower endothelial protective function. We aimed to investigate whether type 2 diabetes impairs HDL endothelium protective functions in people with otherwise normal lipid profile.

**Methods:**

In a case-control study (n = 41 per group) nested in the Cooper Center Longitudinal Study we tested the ability of HDL to protect endothelium by stimulating endothelial nitric oxide synthase activity and suppressing NFκB-mediated inflammatory response in endothelial cells. In parallel we measured HDL protein composition, sphinogosine-1-phosphate and P-selectin.

**Results:**

Despite similar levels of plasma HDL-C the HDL in individuals with type 2 diabetes lost almost 40% of its ability to stimulate eNOS activity (*P*<0.001) and 20% of its ability to suppress TNFα-dependent NFκB-mediated inflammatory response in endothelial cells (*P*<0.001) compared to non-T2D controls despite similar BMI and lipid profile (HDL-C, LDL-C, TC, TG). Significantly, the ability of HDL to stimulate eNOS activity was negatively associated with plasma levels of P-selectin, an established marker of endothelial dysfunction (r = −0.32, *P*<0.001). Furthermore, sphingosine-1-phosphate (S1P) levels were decreased in diabetic plasma (*P* = 0.017) and correlated with HDL-mediated eNOS activation.

**Conclusions/Interpretations:**

Collectively, our data suggest that HDL in individuals with type 2 diabetes loses its ability to maintain proper endothelial function independent of HDL-C, perhaps due to loss of S1P, and may contribute to development of diabetic complications.

## Introduction

Although dyslipidemia characterized by high triglycerides and low levels of HDL-cholesterol (HDL-C) is a hallmark of diabetes, mounting evidence supports the idea that HDL function rather than HDL-C concentration is the relevant measure of HDL protective properties. Three studies have demonstrated that HDL cholesterol efflux capacity (CEC), the ability to accept cholesterol from lipid-loaded macrophages, is impaired in people with cardiovascular disease and is inversely related to risk of CVD.[1–3] In addition to the reverse cholesterol transport from peripheral tissues, several other biological functions of HDL have been identified by which HDL exerts protective effects on endothelial function: namely its ability to reduce inflammation, apoptosis, and thrombosis.[4–6] In healthy individuals, HDL is anti-inflammatory; however, in chronic illnesses characterized by systemic inflammation, such as diabetes, HDL may become “dysfunctional” and actually promote inflammatory responses.[7, 8]

Studies of HDL endothelial protective functions suggest that endothelial and vascular effects of HDL are heterogeneous and may be impaired in many disease states such coronary artery disease, diabetes, and chronic kidney studies.[4, 9, 10] In particular HDL may protect endothelium through its ability to stimulate nitric oxide production by endothelial cells.[4, 11] Ex vivo studies of endothelial cells incubated with isolated HDL from healthy volunteers demonstrate that HDL activates endothelial nitric oxide synthase (eNOS) and stimulates NO production.[12] Furthermore, infusion of reconstituted HDL in patients with type 2 diabetes (T2D) restores endothelial function as measured by forearm blood flow responses.[13] The ability to stimulate eNOS activity and NO production, however, may not be preserved in HDL from obese people with T2D and metabolic syndrome (low HDL, high triglycerides, and large waist circumference).[14]

HDL activates eNOS in cultured endothelial cells *in vitro*, however, the specific molecular mechanism of the activation is not clear. The sphigosine-1-phosphate (S1P), a bioactive phospholipid primarily carried in plasma on HDL, can directly interact with endothelial S1P receptors, activating Akt and eNOS.[15] In agreement with this mechanism, HDL had no effect upon eNOS activation and NO production in mice lacking S1P receptors.[15] Other studies suggest that apoA-I, the main HDL protein, may be able to activate eNOS through its interaction with SR-BI on endothelial cells, and mice lacking SR-BI fail to stimulate eNOS and produce NO in response to HDL stimulation.[12] Furthermore, we have demonstrated previously that isolated HDL from humans increases eNOS activity and reduces endothelial inflammation in primary endothelial cells.[8] Finally, in recent studies, cholesterol efflux to HDL from endothelial cells via the ATP-binding transporter ABCG1 has also been shown to maintain endothelial function in mice fed a high cholesterol diet (a model known to induce endothelial dysfunction)[16] and in humans with coronary endothelial dysfunction.[17] Collectively, these studies suggest that HDL may play an important role in maintaining proper endothelial function, and that HDL dysfunction may contribute to endothelial dysfunction.

In this study, we aimed to determine whether T2D, in people with otherwise apparently normal lipid profiles, may impair HDL protective function of endothelial cells as assessed by ability of HDL to stimulate eNOS Ser1177 phosphorylation and/or inhibit TNF-α dependent NF-κB activation, and thus contribute to endothelial dysfunction associated with T2D. Our results show that HDL in people with T2D loses its endothelium protective properties independent of HDL-C levels and the loss of ability to stimulate eNOS activation is associated with decreased levels of S1P and increased circulating P-selectin, a marker of endothelial dysfunction. Moreover, the loss of HDL function was independent of the level of glycemic control.

## Materials and methods

### Study population

We obtained plasma samples from community dwelling participants from the Cooper Center Longitudinal Study (CCLS). The CCLS is an observational database of patient visits to the Cooper Clinic in Dallas, Texas, a preventive medicine practice established in 1970 which began collecting blood samples from participants in 1999. CCLS participants are generally healthy and are either self-referred or referred by their employers for preventive health examination which include a standardized medical examination, anthropometric measurements, fasting laboratory studies, and a maximal treadmill exercise test. Participants provided informed consent for the use of their data for research purposes. The CCLS database is maintained by The Cooper Institute, a nonprofit independent research institute and privacy precautions were maintained through institute policy. The data collection and informed consent processes were reviewed and approved by the IRB at The Cooper Institute.

The sub-sample of the CCLS utilized for this study was seen after 2008 during which 11,838 unique individuals had at least one visit with stored plasma acquisition in addition to the preventive medical examination. Key clinical variables included height and weight measured using a standard clinical stadiometer and scale to calculate body mass index and seated resting blood pressure measured with a calibrated sphygmomanometer. Laboratory studies obtained after a 12-hour fast included blood glucose, hemoglobin A1c, and cholesterol profile. Plasma samples were stored in 1.5 milliliter vials at -70°C and were not previously thawed.

The CCLS database was queried for all patients with available plasma and a diagnosis of type 2 diabetes, as defined by: self-report, blood glucose over 126 mg/dL, use of diabetic medication, or hemoglobin A1c of greater that 6.5% and 349 subjects with T2D were identified. Preliminary data suggested effect size approximately 0.73. We calculated that with n = 31 per group we will achieve 80% power to observe significant difference between people with diabetes and controls at significance level alpha = 0.05. We increased likelihood of significant findings by increasing power to 90% (n = 41) and randomly selected 41 patients with type 2 diabetes and 41 non-diabetic controls matched by age, sex, and BMI.

### Cell-based assays

Primary human microvascular endothelial cells (HMEC) and primary bovine aortic endothelial cells (BAEC) (Invitrogen-Cascade Biological, Carlsbad, CA) were used for the studies. HMEC were used for the p-65 NFκB assay, and the BAEC were used in the eNOS stimulation assays as they proved superior in cell batch-to-batch reproducibility. In preliminary experiments of eNOS activation assay we used bovine aortic endothelial cells (BAEC) and in human aortic endothelial cells (HAEC). The control HDL isolated from apparently healthy people robustly stimulated eNOS Ser1177 phosphorylation compared to untreated cells (1.4x fold induction with 50 μg/mL HDL and maximal 1.6 and 1.7x induction with 100 nM insulin in HAEC and BAEC cell lines, respectively). Because BAEC cells responded to HDL in identical fashion as HAEC cells and provided more reproducible response to positive control, we used BAEC cells for all our eNOS experiments. Similarly, in preliminary experiments of HDL anti-inflammatory assay the HMEC cells provided similar response, but superior robustness to HAEC cells and therefore we used HMEC cells for our studies. Both cell types were cultured in RPMI 1640 supplemented with 10% fetal bovine serum (Hyclone Laboratories, Logan, UT) and 12 μg/mL of bovine brain extract (Clonetics, Walkersville, MD), L-glutamine (2 mM), sodium pyruvate (1 mM) and nonessential amino acids in the presence of penicillin (100 units/mL) and maintained at 37°C in 5% CO_2_. For the assays HMEC and BAEC were plated in 24-well plates at 37°C in 5% CO_2_. For the anti-inflammatory assay the HMEC cells were then incubated with or without 50 μg/mL HDL in HMEC cell culture media (0.1% FBS) for 16h. The HMEC cells were washed extensively with PBS, and then incubated with an inflammatory stimulus (i.e. 5 ng/mL TNFα for 4 h) and cell lysates were harvested in the presence of protease and phosphatase inhibitors for analysis of phosphorylation of p65 (a marker of NFκB activation). For the eNOS activation assay the BAEC cells were treated with 50 μg/mL HDL for 30 min [18] and cell lysates were harvested in the presence of protease and phosphatase inhibitors. The cell lysates were assayed for phospho-Ser1179 eNOS (equivalent to human Ser1177) by Western blot analysis.

For the analysis the clinical samples were randomized and laboratory staff performing the analysis was blinded to the sample identity. Assay was performed in 24-well plates with all samples analyzed in parallel in a single batch as described above. For Western blot analysis the SDS-PAGE electrophoresis was performed using a 4–12% gradient gels, transferred to membranes and stained with specific antibodies (see below). The blots were scanned and the densitometry measurements from each gel were first normalized to a pooled normal HDL which was included in a random position on every gel to correct for gel-to-gel variability. Subsequently, for each sample, the signal of phosphorylated protein was normalized to total eNOS and p65, respectively. To calculate % activation the corrected and normalized data was then normalized to the values obtained from control samples incubated with vehicle alone (or TNF-alpha treated for the anti-inflammatory activity), and percent change relative to the control treatment condition was calculated.

Anti-phospho-p65 (#3033), anti-phospho-eNOS (Ser1177; #9570) (cross-reactive to bovine Ser1179 phospho-eNOS), anti-total p65 and eNOS were from Cell Signaling (Beverly, MA). Insulin levels were determined by ELISA kit (Crystal Chem, Inc. Downers Grove Il).

### P-selectin

Plasma levels of P-selectin were measured by ELISA (R&D Systems, MN) according to manufacturer protocol.

### HDL isolation

HDL was isolated from EDTA-anticoagulated plasma, using sequential ultracentrifugation (d = 1.063–1.21 mg/mL) essentially as described previously[19] in table-top ultracentrifuge Beckman Optima XL with TL100.1 rotor (Beckman, CA). Aliquots of HDL were immediately frozen and stored at -80 C prior to analysis as it was previously shown not to impact HDL protective effects on endothelial cells.[20]

### Protein digestion

HDL (10 μg protein) was solubilized with 0.1% RapiGest (Waters, MA) in 200 mM ammonium bicarbonate, spiked with 1 μg of ^15^N-apoA-I as internal standard,[21] reduced with dithiothreitol, alkylated with iodoacetamide, and digested with two additions of trypsin (1:20, w/w HDL protein; Promega, WI) initially for 3 h at 37°C, and after second addition overnight. After acidic hydrolysis of RapiGest, samples were dried, and stored at −20°C until analysis. Samples were reconstituted in 5% acetonitrile, 0.1% formic acid to 0.25 μg/μL.[22]

### Selection of the peptides and transitions for SRM analysis

At least two peptides for each protein were chosen for SRM analysis based on spectral count in shotgun experiments, observed frequency in the PeptideAtlas mass spectral database, and on screening experiments that evaluate the correlation of peptides from the same protein with one another across clinical study populations.[21] Peptides containing methionine residues were excluded from consideration. The transitions monitored for each peptide were selected based on signal intensity from a screening experiment. List of peptides and acquisition conditions is provided in S1 Table (isoforms of SAA, SAA1 and SAA2, were not distinguished and are reported together as SAA1/2).

### Selected-reaction monitoring analysis

Tryptic digests of HDL were chromatographed using a nanoAquity UPLC (Waters, MA) with a C18 trapping column (XBridge BEH C18 100 Å, 5 μm, 0.1 x 40 mm, Waters, MA) (trapping flow rate 4 μL/min), a capillary XBridge BEH C18 analytical column (XBridge BEH C18 100Å, 3.5μm, 100x0.075 mm, Waters, MA), and a 30 min linear gradient of acetonitrile, 0.1% formic acid (7–35%) in 0.1% formic acid in water at a flow rate of 0.6 μL/min. The NanoAquity UPLC was connected to a Thermo TSQ Vantage triple-quadrupole mass spectrometer with electrospray ionization. The instrument was operated in SRM mode with 10 ms dwell time and the peptides were monitored collision energies optimized to maximize the signal. Peak areas were integrated using Skyline software.[23]

### Measurement of S1P

Sphingosine-1-phosphate was extracted from 10 μL of plasma after dilution with 50μL with 30mM citric Acid/40mM sodium phosphate buffer pH 4.0 and spiking with 5uL internal standard (S1P-C17 base, Avanti LM-6002) to a concentration of 0.23μM.[24] After thorough mixing the sample was extracted with 275 μL of 1-butanol (Fisher A399), spun down and 220 μL were collected and dried down. The dried down samples were reconstituted for LCMS analysis in 125 μL of 50% methanol, 1% formic acid in 5mM ammonium formate. Samples were spun down and 10μL injected onto LCMS consisting of Shimadzu Prominence LC system and 4000 QTrap (ABSciex) in Turbo Ion Spray Mode. Separation was accomplished on a Luna 3μm C18 100Å 50x2.0mm column (Phenomenex) with 1.5 minute gradient from LC buffer A (50% methanol, 1% formic acid in 5mM ammonium formate) to LC buffer B (89% methanol, 10% 2−propanol, 1% formic acid in 5mM ammonium formate) and S1P was quantified together with the internal standard (S1P-C17) using multiple-reaction-monitoring. Calibration was accomplished using a single point calibrator repeatedly assayed throughout the analysis.

### Statistical analysis

All HDL functional assays were performed in duplicate and the mean was computed and used for analysis. Triglyceride concentrations were log-transformed. Proteomics peptide measurements were normalized to ^15^N-apoA-I peptide VQPYLDDFQK and response of two peptides for each protein was averaged, log2 transformed and standardized prior to further analysis. Four samples failed the LCMS analysis and therefore were not used in the data analysis. Outliers in the data were identified using Median Absolute Deviation (MAD) approach[25] as data points exceeding 3*MAD from a median of a given group and were eliminated from corresponding analysis. Group comparisons were performed using Student’s t-test (non-parametric Mann-Whitney U test for proteomics) and associations between continuous variables were established by using linear regression and Pearson’s correlation coefficient, association with diabetes status were established using logistic regression. Multivariate logistic regression was used to investigate associations between diabetes status and HDL function controlling for potential confounders. Statistical significance was determined for *P* < 0.05 for all tests. Statistical analyses were performed using R software (version. 3.1).

## Results

All subjects in the present study were moderately overweight (BMI>25, mean BMI 29.3±4.6 kg/m^2^). Consistent with their diabetes diagnosis, the subjects in the diabetic group had modest but significantly higher blood glucose and glycated hemoglobin A1c levels (Table 1). There were no differences between control and diabetic groups in any of the plasma lipids measures including HDL-C, LDL-C and triglycerides, as well as in blood pressure, or systemic measure of inflammation (hsCRP). Clinical and demographic characteristics of the study population are presented in the Table 1.

**Table 1:** Clinical characteristics of the study population.

	Control subjects (n = 41)	T2D subjects (n = 41)	*P*
Age (yr)	59.1 ± 7.4	60.1 ± 7.3	0.52
Sex (F/M)	7/34	7/34	
Body mass index, kg/m^2^	29.1 ± 4.5	29.5 ± 4.7	0.71
Fasting glucose, mg/dL	96.5 ± 9.7	132 ± 30	<0.001
Hemoglobin A1C, % (mmol/mol)	5.6 ± 0.4 (38 ± 4.4)	6.8 ± 0.9 (51.0 ± 9.8)	<0.001
high sensitivity CRP, ng/mL	2.6 ± 6.7	4.5 ± 5.6	0.16
HDL cholesterol, mg/dL	54 ± 15.8	53.7 ± 15	0.95
LDL cholesterol, mg/dL	94.8 ± 34.9	88.9 ± 38.9	0.47
Total cholesterol, mg/dL	172 ± 40.6	166.6 ± 45.2	0.57
Triglycerides, mg/dL	116.2 ± 49.4	119.8 ± 46.2	0.74
Mean arterial pressure, mm Hg	93 ± 8.1	92.9 ± 13.1	0.97

Values are presented as mean ± SD or number of subjects

### HDL in type 2 diabetes loses anti-inflammatory properties

**Fig 1:**
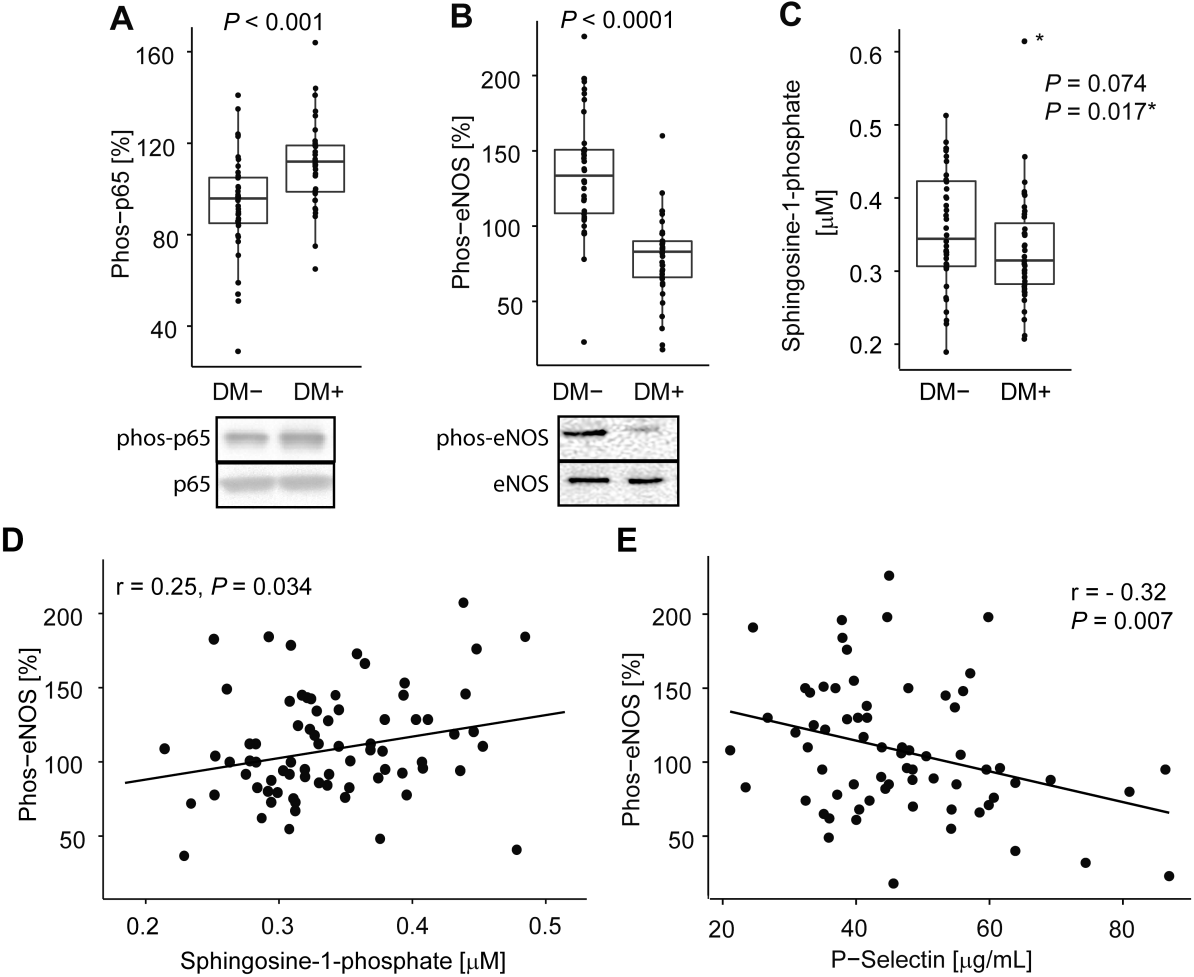
Protective effects of HDL on endothelial cells are impaired in diabetes, correlate with S1P and negatively associate with *in vivo* measure of endothelial dysfunction. **(A)** The ability of HDL to suppress NFκB activation was measured as phosphorylation of p65 in HMEC after 16 h incubation with HDL (50 μg/mL) followed by 4 h stimulation with TNFα (n = 41 per group). **(B)** The ability of HDL to stimulate eNOS Ser1179 phosphorylation was measured in BAEC after 30 min incubation with HDL (50 μg/mL) (n = 38 non-diabetic, n = 41 diabetic subjects). (Data is expressed relative to cells not treated with HDL). **(C)** Sphigosine-1-phosphate concentration measured by LC-MS is reduced in patients with diabetes and **(D)** correlates positively with HDL ability to stimulate eNOS phosphorylation (n = 40 non-diabetic, n = 39 diabetic subjects; *excluded outlier and *P*-value after exclusion). **(E)** The ability of HDL to stimulate eNOS is inversely correlated negatively with level of P-selectin in plasma, an *in vivo* measure of endothelial dysfunction (Pearson correlation coefficient; n = 81 after an outlier exclusion).

HDL has been shown to exert anti-inflammatory effects on endothelial cells suppressing activation of NFκB in human endothelial cells,[8] however, this function may be impaired in people with T2D. As a marker of NFκB activation we measured TNFα-dependent phosphorylation of p65, a key event in the NFκB pathway which is necessary for NFκB translocation and cellular inflammatory response,[26, 27] after pretreatment with HDL. When incubated with human endothelial cells, the HDL isolated from the non-diabetic controls suppressed the TNFα dependent NFκB activation in endothelial cells (mean = 93.2±22.4%; n = 41) (Fig 1A). In contrast, HDL from age and sex matched diabetic patients was unable to suppress the NFκB activation in these cells and even displayed pro-inflammatory effects (mean = 111.0±19.0%, n = 41).

### HDL in diabetes loses ability to stimulate eNOS activation

Diabetes is associated with endothelial dysfunction and HDL can protect endothelium by stimulating production of nitric oxide through activation of eNOS. We therefore measured the ability of HDL to stimulate phosphorylation of eNOS Ser1177, a marker of activation of endothelial nitric oxide synthase and NO production in endothelial cells.[28] Compared to the HDL from non-diabetic subjects, the HDL from the diabetic subjects lost its ability to stimulate Ser1179 phosphorylation and eNOS activation eliciting 40% less activity (*P*<0.0001) (Fig 1B). Notably, the response to the HDL from people with T2D was less than that of an untreated control, suggesting a suppressive rather than stimulatory effect.

### Endothelial functions of HDL are independent of dyslipidemia and glycemic control

**Fig 2:**
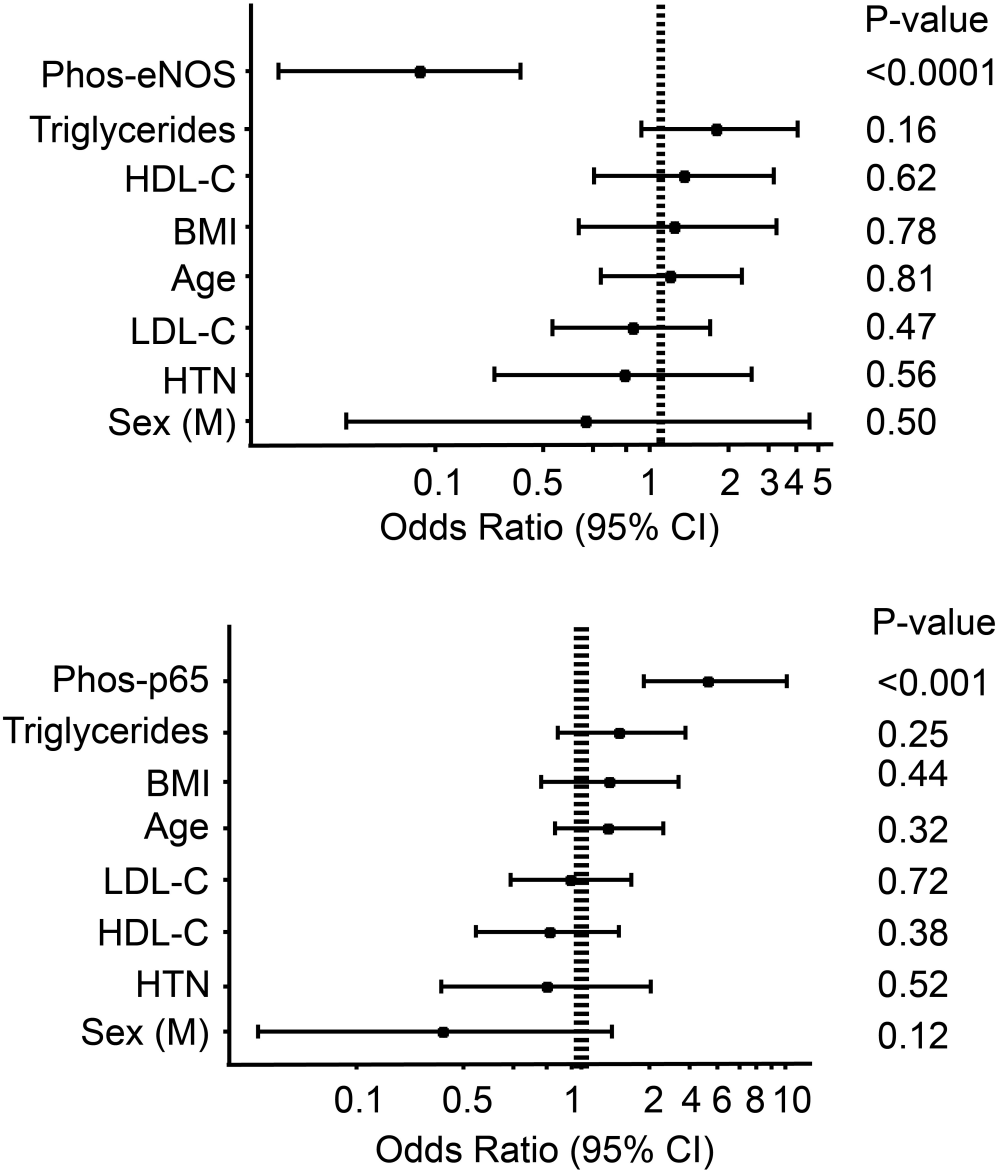
Odds ratios for type 2 diabetes according to HDL endothelial protective functions and selected dyslipidemia and common risk factors. Multivariate logistic regression models included the listed dyslipidemia and other cardiovascular risk factors and odds ratios for continuous variables are presented per 1-SD increase for **(A)** HDL ability to stimulate eNOS activation, and **(B)** for the suppression of NFκB activation.

To further investigate factors, which may be associated with the impaired HDL functionality in diabetic patients, we employed multivariate logistic regression models. Both HDL anti-inflammatory activity (as measured by ability to suppress p65 phosphorylation) and stimulation of eNOS activity were independent of age, sex, and HDL-C (OR = 2.8, 95%CI 1.6–5.5, *P* = 0.001, and OR = 0.08, 95%CI 0.02–0.2, *P*<0.001, Table 2). The endothelium protective functions of HDL remained significantly associated with diabetes status even after adjusting for fasting glucose or glycated HbA1c (Table 2). In models with selected cardiovascular factors age, LDL-C, HDL-C, triglycerides or BMI only the HDL endothelium protective functions were strongly associated with the diabetes status (Fig 2). Moreover, linear regression analysis of HDL activity versus fasting glucose or glycated HbA1c, while revealing strong negative correlation of phos-eNOS (r = −0.32, *P* = 0.01, r = −0.35, *P* = 0.01 for glucose and HbA1c, respectively) explained only 12% of the HDL activity variance. The correlation with HDL anti-inflammatory properties was even weaker (r = 0.2, *P* = N.S., r = 0.25, *P* = 0.01 for fasting glucose and glycated HbA1c, respectively).

**Table 2:** Multivariate logistic regression analysis of diabetic status as a function of HDL activity adjusted for various potential confounders and measures of diabetes.

	Model	Tested variable	OR	95% CI	*P*
Univariate Models	Glucose	Glucose	1.16	1.1–1.24	<0.001
	HbA1c	HbA1c	51.61	11.45–424.9	<0.001
	phos-p65^#^	phos-p65	2.78	1.59–5.46	0.001
	phos-eNOS^$^	phos-eNOS	0.07	0.02–0.2	<0.001
Multivariate models—age, sex and HDL-C	phos-p65+age+sex	phos-p65	2.76	1.57–5.45	0.001
	phos-p65+age+sex+HDL-C	phos-p65	2.78	1.59–5.48	0.001
	phos-eNOS+age+sex	phos-eNOS	0.07	0.02–0.21	<0.001
	phos-eNOS+age+sex+HDL-C	phos-eNOS	0.07	0.02–0.21	<0.001
Multivariate models—age, sex and glucose or HbA1c	phos-p65+age+sex+glucose	phos-p65	7.31	2.45–36.97	0.003
	phos-p65+age+sex+HbA1c	phos-p65	3.10	1.26–9.25	0.02
	phos-eNOS+age+sex+glucose	phos-eNOS	0.03	0–0.19	0.002
	phos-eNOS+age+sex+HbA1c	phos-eNOS	0.06	0.01–0.24	0.001

Odds ratio of diabetic status for 1-SD of HDL activity, or for 1 unit of fasting glucose or HbA1c

^#^—phos-p65—HDL anti-inflammatory activity

^$^—phos-eNOS—HDL eNOS stimulating activity

### S1P levels are decreased in diabetes and associate with HDL function

Previous studies suggested that HDL effects on endothelial cells may be mediated at least in part through sphingosine-1-phosphate (S1P), for which HDL is the major carrier in plasma (over 70% of plasma levels of S1P are associated with HDL).[15] We therefore measured plasma levels of S1P. The S1P levels were 10% lower in diabetes compared to controls (*P* = 0.074, after excluding the single outlier in diabetic group, *P* = 0.017) (Fig 1C). Moreover, S1P levels were strongly associated with the ability of HDL to stimulate eNOS activity (r = 0.22, *P* = 0.055, r = 0.25, *P* = 0.034 after excluding a single outlier) (Fig 1D). Lack of association of the outlier S1P level with any other measures suggest that the high S1P value is likely due to assay technical issues. No association of S1P with HDL anti-inflammatory activity was observed.

### The eNOS stimulation by HDL is negatively associated with endothelial dysfunction

Because our data implicate that diabetes impairs the ability of HDL to protect the endothelium, we investigated whether HDL function is associated with endothelial dysfunction *in vivo*. As an *in vivo* measure of endothelial dysfunction we quantified circulating levels of P-selectin, an established marker of endothelial dysfunction.[29] P-selectin was moderately elevated in diabetic patients (11% increase compared to non-diabetic patients, *P* = 0.14; 15% increase, *P* = 0.04 after excluding a single non-diabetic subject with extremely high P-selectin levels). Moreover, the ability of HDL to stimulate eNOS was negatively associated with the plasma P-selectin concentration (r = −0.32, *P* = 0.007) (Fig 1E).

### HDL protein composition in type 2 diabetes

To investigate if HDL protein composition was associated with the loss of the protective properties of HDL, we employed the quantitative targeted proteomics approach that we have developed previously.[21] We quantified 36 major HDL associated proteins (Table 3) using ^15^N-isotope labeled apolipoprotein A-I for internal standardization and compared the relative abundance of these proteins between diabetic and non-diabetic subjects. While several proteins were significantly different between diabetic subjects and non-diabetic controls in univariate analysis, apoC-III (*P* = 0.03), SAA1/2 (SAA1 and SAA2 measured together) (*P* = 0.0032) increased and apoA-IV (*P* = 0.034) decreased, after controlling for multiple comparisons, none of the protein differences remained significant. Moreover, only SAA1/2 showed modest inverse correlation with HDL eNOS stimulating activity suggesting that it may in part contribute to the impairment of this HDL function in T2D (r = −0.26, *P* = 0.026). Collectively, our proteomics data indicate that HDL protein composition does not significantly contribute to HDL functional impairment in people with T2D.

**Table 3:** Protein quantified in the targeted proteomics analysis.

Protein	Protein Name	Protein	Protein Name
ALB	Serum albumin	CETP	Cholesteryl ester transfer protein
APOA1	Apolipoprotein A-I	CLU	Clusterin(Apolipoprotein J)
APOA2	Apolipoprotein A-II	HPR	Haptoglobin-related protein
APOA4	Apolipoprotein A-IV	HPX	Hemopexin (Beta-1B-glycoprotein)
APOB	Apolipoprotein B-100	LCAT	Lecithin-cholesterol acyltransferase
APOC1	Apolipoprotein C-I	LPA	Apolipoprotein(a)
APOC2	Apolipoprotein C-II	PCYOX1	Prenylcysteine oxidase 1
APOC3	Apolipoprotein C-III	PLTP	Phospholipid transfer protein
APOC4	Apolipoprotein C-IV	PON1	Serum paraoxonase/arylesterase 1
APOD	Apolipoprotein D	PON3	Serum paraoxonase/lactonase 3
APOE	Apolipoprotein E	RBP4	Retinol-binding protein 4
APOF	Apolipoprotein F	SAA1_2	Serum Amyloid A-1/Serum Amyloid-2 proteins
APOH	Beta-2-glycoprotein 1	SAA4	Serum amyloid A-4 protein
APOL1	Apolipoprotein L1	SERPINA1	Alpha-1-antitrypsin
APOM	Apolipoprotein M	SERPINA4	Kallistatin (Kallikrein inhibitor)
C3	Complement C3	VDBP	Vitamin D Binding Protein
C4A	Complement C4-A	VTN	Vitronectin

## Discussion

In the present study of community dwelling patients with T2D and normal lipid profiles, we found that diabetes significantly impaired HDL endothelial protective functions, as measured by ability of HDL to stimulate eNOS and attenuate NF-κB activation in response to TNFα, despite lack of differences, compared to non-diabetic subjects, in traditional clinical measures associated with metabolic syndrome and cardiovascular risk (HDL-C, TG, LDL-C, BMI, hypertension or CRP). Moreover, our results strongly suggest that *in vivo* endothelial dysfunction may be associated with the observed HDL dysfunction and that this HDL dysfunction is not associated with level of glycemic control. Collectively, our data indicate that the ability of HDL to maintain endothelial health is impaired in T2D and may contribute the endothelial dysfunction associated with T2D.

Multiple studies showed that diabetes is associated with endothelial dysfunction and that endothelial dysfunction may be associated with insulin resistance and precede diabetes onset[29–31] and HDL has been shown to stimulate eNOS/NO mediated vasodilation[14, 16] as well as suppress the endothelial inflammatory response to TNFα[32] and decrease endothelial cell exocytosis.[33] Elevation of HDL by infusion of reconstituted HDL increased endothelial function as measured by increased blood flow in response to acetylcholine,[13] improved flow-mediated vasodilation in type 2 diabetic patients[34] and the improved anti-inflammatory function of HDL was strongly correlated with elevation of apoA-I and HDL-C concentration.[35]

In a recent study the ability of HDL to protect the endothelium was impaired in people with T2D and metabolic syndrome (markedly low HDL, 33 vs. 53 mg/dL in controls; higher central obesity waist circumference 116 vs 91 cm, BMI 33 vs 27 kg/m^2^; and elevated triglycerides 225 vs 168 mg/dL),[14] raising the question whether the observed phenomena is merely reflection of low HDL-C levels. HDL inflammatory index (ability to protect from oxidized-LDL induced inflammation) was also reduced in obese, hypertensive subjects with previous CVD compared to healthy controls.[36] In contrast, our results clearly demonstrate that the HDL dysfunction associated with diabetes is independent of HDL-C levels or any other indicators of metabolic syndrome or other co-morbidities (Fig 2). Several studies suggest that the impairment of the HDL function may be associated with glycation of apoA-I.[37] In the current study, the HDL eNOS activity was only very modestly correlated with fasting glucose or glycated HbA1c and independent of it in multivariate regression models (Table 2), suggesting that the ability of the HDL to stimulate eNOS activity is also independent of glycemic control.

The ability of HDL to directly activate eNOS in primary aortic endothelial cells may be mediated by several mechanisms. Multiple lines of evidence suggest sphingosine-1-phosphate (S1P), primarily carried in plasma in HDL,[38] is one of the key effectors of HDL protective endothelial functions.[39–41] S1P can directly interact with endothelial S1P receptors (S1PR1, S1PR3), activating Akt and eNOS.[15] In the current study we found decreased levels of S1P in diabetic patients and a strong negative correlation between plasma S1P levels and ability of HDL to activate eNOS. In agreement with our results, a recent study in mouse models of diabetes showed decrease of plasma S1P levels in streptozotocin-induced diabetic mice.[42] Notably, S1P content of HDL has been associated with the presence of apoM.[43] However, we found that apoM levels were not different between T2D patients and controls suggesting that while apoM may control a large portion of HDL S1P content, it is not associated with decrease of S1P in diabetes and ultimately for HDL functionality. Indeed, a recent study showed that HDL may be replenished with S1P, even in the absence of apoM, completely restoring its eNOS stimulating activity.[44]

In addition to actions of HDL-carried S1P, apoA-I has also been reported to activate eNOS through its interaction with SR-BI on endothelial cells.[45] However, others have found that despite being the ligand for SR-BI, lipid free apoA-I failed to activate eNOS[46] suggesting that other HDL components may be important or are required to mediate the apoA-I effect and allow it to interact with SR-BI. While our proteomics analysis did not detect a significant difference in HDL-associated apoA-I levels between T2D and controls, other investigators have suggested that hyperglycemia results in nonenzymatic glycation of apoA-I[37] and that glycation of apoA-I, and advanced glycation end-products (AGEs) in general, may contribute to reduction of HDL ability to activate eNOS.[47] Although the T2D patients had elevated glucose and glycated HbA1c, the concentrations we observed only slightly correlated with the ability of HDL to activate eNOS. Multivariate regression suggested that the inability of HDL from people with T2D to activate eNOS is independent of glucose or hemoglobin A1c concentrations, indicating that glycation may contribute only partially to the diabetes associated HDL dysfunction.

The heterogeneity of the vascular effects of HDL may be attributed to HDL-associated proteome and lipid content as well as post-translational protein modification. The total pool of HDL within an individual is composed of numerous HDL subpopulations with distinct protein and lipid composition. Although proteomic studies have reported >90 HDL associated proteins, only a small fraction of these has been consistently found associated with HDL. We performed quantitative analysis of 35 proteins consistently associated with HDL including proteins with relative abundance as low as 0.1% of the abundance of apoA-I, and did not find any significant differences between T2D and control patients. These results suggest that differences in other, low abundance proteins not captured in our analysis and/or post translational modification of the measured proteins may be responsible for functional differences. HDL also carries a rich ensemble of phospholipids in addition to S1P, which may mediate HDL function and have been associated with insulin resistance and diabetes.[48] It is thus possible that other lipids then S1P may contribute to the HDL dysfunction in diabetes.

In future studies, it will be important to determine whether specific post-translational modifications and lipid components of HDL mediate the impairment of HDL endothelial protective function in diabetes and whether and how therapies targeting HDL affect the HDL ability to protect endothelium.

Strengths of our study include the unique patient population, the similar baseline characteristics of control and diabetic subjects, and the use of novel assays for quantifying HDL endothelial protective functions. Limitations include the lack direct measurement of *in vivo* endothelial function, S1P levels in HDL and data on HDL protein glycation or other post-translation modifications.

## Conclusions

In summary, our study provided strong evidence suggesting that type 2 diabetes impairs HDL-mediated eNOS activation and HDL-mediated attenuation of NFκB signaling in endothelial cells and that this HDL dysfunction may be associated with *in vivo* endothelial dysfunction. This HDL dysfunction appears to be independent of plasma lipids (HDL-C, TG, LDL-C) or other risk factors associated with endothelial dysfunction (i.e. BMI or central obesity). These findings suggest that loss of HDL endothelial protective functions may contribute to increased risk of CVD in diabetes. Our findings also suggest that these HDL functions might be target for development new therapeutics for CVD.

## Supporting information

S1 Table List of proteins and corresponding peptides included in targeted proteomics analysis. (PDF)
