# Supplementary material for: Type 2 diabetes is associated with loss of HDL endothelium protective functions
Source: PLoS One. 2018 Mar 15;13(3):e0192616. doi: 10.1371/journal.pone.0192616 (PMC5854245; doi:10.1371/journal.pone.0192616)
Supplement: S1 Table — (PDF) [file pone.0192616.s001.pdf]

Supplementary Table I. List of proteins and corresponding peptides included in targeted proteomics analysis.

Protein	Peptide	Precursor Mz	Precursor Charge	Collision Energy [NCE]
ALB	FQNALLVR	480.78	2	15
ALB	LVNEVTEFAK	575.31	2	18
APOA1	DYVSQFEGSALGK	700.84	2	22
APOA1_15N	DYVSQFEGSALGK	708.32	2	22
APOA1	VQPYLDDFQK	626.81	2	19
APOA1_15N	VQPYLDDFQK	633.29	2	19
APOA2	EQLTPLIK	471.29	2	15
APOA2	SPELQAEAK	486.75	2	15
APOA4	LGEVNTYAGDLQK	704.36	2	22
APOA4	LTPYADEFK	542.27	2	17
APOB	IEIPLPFGGK	535.82	2	17
APOB	SVSLPSLDPASAK	636.35	2	20
APOC1	EFGNTLEDK	526.75	2	17
APOC1	EWFSETFQK	601.28	2	19
APOC2	TAAQNLYEK	519.27	2	16
APOC2	TYLPAVDEK	518.27	2	16
APOC3	DALSSVQESQVAQQAR	858.93	2	26
APOC3	DYWSTVK	449.72	2	14
APOC4	AWFLESK	440.73	2	14
APOC4	ELLETVVNR	536.80	2	17
APOD	NPNLPPETVDSLK	712.38	2	22
APOD	VLNQELR	436.25	2	14
APOE	AATVGSLAGQPLQER	749.40	2	23
APOE	SELEEQLTPVAEETR	865.93	2	26
APOF	SGVQQLIQYYQDQK	849.43	2	26
APOF	SLPTEDC[+57]ENEK	661.28	2	20
APOH	ATVVYQGER	511.77	2	16
APOH	TC[+57]PKPDDLPFSTVVPLK	638.67	3	27
APOL1	LNILNNNYK	553.30	2	17
APOL1	VTEPISAESGEQVER	815.90	2	25
APOM	AFLLTPR	409.25	2	13
APOM	SLTSC[+57]LDSK	505.74	2	16
C3	TGLQEVEVK	501.78	2	16
C3	TIYTPGSTVLYR	685.87	2	21
C4A	GSFEFPVGDAVSK	670.33	2	21
C4A	VFALDQK	410.73	2	13
CETP	ASYPDITGEK	540.76	2	17
CETP	VIQTAFQR	481.77	2	15
CLU	ELDESLQVAER	644.82	2	20
CLU	LFDSDPITVTVPVEVSR	937.50	2	28
HPR	LPEC[+57]EAVC[+57]GKPK	694.34	2	21
HPR	VVLHPNYHQVDIGLIK	615.68	3	26

LCAT	SSGLVSNAPGVQIR	692.88	2	21
LCAT	STELC[+57]GLWQGR	653.81	2	20
LPA	GTLSTTITGR	503.78	2	16
LPA	TPAYYPNAGLIK	654.35	2	20
PCYOX1	LFLSYDYAVK	609.82	2	19
PCYOX1	LVC[+57]SGLLQASK	588.33	2	18
PLTP	FLEQELETITIPDLR	908.99	2	28
PLTP	GAFFPLTER	519.27	2	16
PON1	IQNILTEEPK	592.83	2	19
PON1	STVELFK	412.23	2	13
PON3	AQALEISGGFDK	618.32	2	19
PON3	LLNYNPEDPPGSEVLR	906.96	2	28
RBP4	LLNLDGTC[+57]ADSYSFVFSR	1032.99	2	31
RBP4	YWGVASFLQK	599.82	2	19
SAA1_2	FFGHGAEDSLADQAANEWGR	1089.49	2	33
SAA1_2	SFFSFLGEAFDGAR	775.87	2	24
SAA4	FRPDGLPK	465.26	2	15
SAA4	GPGGVWAAK	421.73	2	14
SERPINA1	LSITGTYDLK	555.81	2	17
SERPINA1	SVLGQLGITK	508.31	2	16
SERPINA4	FYYLIASETPGK	694.86	2	21
SERPINA4	VGSALFLSHNLK	643.37	2	20
VDBP	HLSLLTTLSNR	627.86	2	20
VDBP	VLEPTLK	400.25	2	13
VTN	DVWGIEGPIDAAFTR	823.91	2	25
VTN	FEDGVLDPDYPR	711.83	2	22
